# Interplay of the heart, spleen, and bone marrow in heart failure: the role of splenic extramedullary hematopoiesis

**DOI:** 10.1007/s10741-024-10418-6

**Published:** 2024-07-10

**Authors:** Hiroaki Hiraiwa, Yoshimitsu Yura, Takahiro Okumura, Toyoaki Murohara

**Affiliations:** Department of Cardiology, Nagoya University Graduate School of Medicine, 65 Tsurumai-cho, Showa-ku, Nagoya, 466-8550 Japan

**Keywords:** Bone marrow, Extramedullary hematopoiesis, Heart failure, HFpEF, Spleen

## Abstract

Improvements in therapies for heart failure with preserved ejection fraction (HFpEF) are crucial for improving patient outcomes and quality of life. Although HFpEF is the predominant heart failure type among older individuals, its prognosis is often poor owing to the lack of effective therapies. The roles of the spleen and bone marrow are often overlooked in the context of HFpEF. Recent studies suggest that the spleen and bone marrow could play key roles in HFpEF, especially in relation to inflammation and immune responses. The bone marrow can increase production of certain immune cells that can migrate to the heart and contribute to disease. The spleen can contribute to immune responses that either protect or exacerbate heart failure. Extramedullary hematopoiesis in the spleen could play a crucial role in HFpEF. Increased metabolic activity in the spleen, immune cell production and mobilization to the heart, and concomitant cytokine production may occur in heart failure. This leads to systemic chronic inflammation, along with an imbalance of immune cells (macrophages) in the heart, resulting in chronic inflammation and progressive fibrosis, potentially leading to decreased cardiac function. The bone marrow and spleen are involved in altered iron metabolism and anemia, which also contribute to HFpEF. This review presents the concept of an interplay between the heart, spleen, and bone marrow in the setting of HFpEF, with a particular focus on extramedullary hematopoiesis in the spleen. The aim of this review is to discern whether the spleen can serve as a new therapeutic target for HFpEF.

## Introduction

An estimated 64 million people experience heart failure worldwide [1]. Heart failure is the leading cause of hospitalization among adults, with 1-year mortality reported to be between 10 and 35% in various population-wide registries [2–4]. Non-communicable diseases such as obesity, hypertension, and diabetes are major risk factors for heart failure [2, 5]. Heart failure can be grouped into three categories based on the percentage of ejection fraction [6]: heart failure with preserved ejection fraction (HFpEF), heart failure with reduced ejection fraction (HFrEF), and heart failure with midrange ejection fraction (HFmrEF) [6, 7]. HFpEF is defined by an ejection fraction > 50% and is characterized by structural and cellular alterations such as cardiomyocyte hypertrophy, fibrosis, and inflammation, resulting in an inability of the left ventricle to relax properly [7, 8]. HFrEF is defined by an ejection fraction < 40% and is characterized by cardiomyocyte loss, resulting in the development of systolic dysfunction, whereby the left ventricle cannot contract properly [8, 9]. HFmrEF is defined as an ejection fraction of 40–50% [10].

Among patients aged > 65 years with heart failure, almost half have HFpEF [1, 5, 11]. A study conducted to examine biomarkers in heart failure found that biomarkers in HFpEF were associated with markers for inflammation, remodeling, angiogenesis, and hematopoiesis [12]. HFpEF is also associated with elevated levels of pro-inflammatory cytokines [13]. While efficient and specific treatments for HFrEF have been developed, treatment options for HFpEF are lacking [14].

Hematopoiesis is defined as the production of all components of blood and plasma, including cells of the immune system [15]. Hematopoiesis occurs within the hematopoietic system, while bone marrow is the main site for hematopoiesis in adults, and growing evidence points to a role for the spleen in the maintenance and differentiation of hematopoietic stem cells [16]. Extramedullary hematopoiesis refers to blood cell production outside the bone marrow, which can occur in the spleen, liver, adipose tissue, and lymph nodes [17]. The spleen plays an important role in filtering blood and regulating immune responses to various agents and contains large pools of undifferentiated monocytes that can undergo splenic hematopoiesis [18].

Altered iron metabolism and anemia can contribute to HFpEF, and iron deficiency is common in heart failure. The threshold for iron deficiency in patients with heart failure is defined as ferritin of < 100 µg/l or ferritin of 100–300 µg/l if transferrin saturation is < 20% [19–21]. This threshold for iron deficiency has been applied in several studies. Jankowska et al. investigated the relationship between iron deficiency and survival in patients with systolic heart failure and reported a prevalence of iron deficiency in patients with heart failure of 37% (in 546 patients) [22]. The PrEP Registry, a prospective observational study conducted by von Haehling et al., assessed the prevalence and clinical impact of iron deficiency and anemia in heart failure patients in Germany and found a prevalence of 42.5% (in 1198 patients) [23]. Iron deficiency can lead to anemia, which is caused by a decrease in iron levels characterized by decreased levels of hematopoiesis in red blood cells (hypochromia) and diminished mean corpuscular volume (microcytosis) [24]. Most iron in the body is bound to hemoglobin in erythrocytes, with the rest being stored in macrophages and hepatocytes [25]. Besides the bone marrow and liver, the spleen participates in iron metabolism, and macrophages in the red pulp of the spleen recycle iron from senescent red blood cells [25]. Anemia often develops in patients with HFpEF. Its prevalence has been reported over a variety of ranges: 21–68% in hospitalized patients, 19–27% in participants of randomized controlled trials, and 30–33% in patients being treated on an outpatient basis [26]. Anemia is classified as hemoglobin < 13.0 g/dl in men and < 12.0 g/dl in women [27]. The origin of anemia in heart failure is multifactorial and includes important factors such as renal dysfunction, hemodilution, chronic inflammation, certain therapeutics (for example, angiotensin-converting enzyme inhibitors, angiotensin receptor blockers, or beta blockers), bone marrow dysfunction, resistance to erythropoietin, and deficiencies of vitamin B12, folic acid, and iron [28–30].

The role of inflammation in both the onset and progression of heart failure is also of great interest. Most major immune cell types in the steady state are found in the heart, including monocytes, macrophages, T cells, neutrophils, B cells, dendritic cells, natural killer cells, and mast cells [31]. The spleen, being the body’s largest secondary immune organ, hosts a wide range of immunological functions besides its roles in hematopoiesis and red blood cell clearance [18].

This review aims to assess the current status of the role played by extramedullary hematopoiesis in the interplay between the heart, spleen, and bone marrow in the context of heart failure, especially HFpEF. Iron metabolism and anemia are also discussed in view of heart failure. Additionally, this review examines hematopoiesis other than that in the bone marrow, specifically extramedullary emergency hematopoiesis in the spleen, which may be involved in the pathogenesis and progression of HFpEF.

## Medullary hematopoiesis and heart failure

There is increasing evidence of a link between medullary hematopoiesis (occurring in the bone marrow) and heart failure. Clinical studies have shown that levels of bone marrow-derived circulating endothelial progenitor cells are associated with cardiovascular risk factors, including increased age, diabetes, smoking, and hypertension [32].

## Clonal hematopoiesis

Clonal hematopoiesis is a pre-leukemic condition in which hematopoietic stem cells with acquired somatic DNA mutations proliferate in the bone marrow, and its frequency has been reported to increase with age [33]. Previously, the pathological significance of clonal hematopoiesis beyond increasing the risk of hematological malignancy was not well understood, but recent epidemiological analyses and animal experiments have revealed that it is a novel risk factor for cardiovascular diseases [34–36]. Carriers of hematopoiesis of indeterminate potential (CHIP) were shown to have a 1.9-fold increased risk of heart disease compared with non-carriers, and somatic mutations in DNA methyltransferase *DNMT3A*, DNA demethylase *TET2*, additional sex combs-like 1 *ASXL1*, and Janus kinase 2 *JAK2* have been associated with ischemic heart disease [34]. Furthermore, it has been reported that clonal hematopoiesis may be involved in the pathogenesis of heart failure of ischemic origin [37]. CHIP driver genes *TET2* and *DNMT3A* have been shown to be significantly associated with the progression and poor prognosis of ischemic heart failure [37]. Subsequent analyses have reported, from an epidemiological perspective, that clonal hematopoiesis is significantly associated with the progression and poor prognosis of HFrEF, regardless of the etiology [38]. In addition, mutations in *TET2* or *DNMT3A* have demonstrated a profoundly increased mortality driven by the progression of heart failure [37]. Furthermore, *TET2* CHIP has been identified as an independent risk factor for HFpEF, independent of conventional cardiovascular risk factors and coronary artery disease [39]. The mechanism by which clonal hematopoiesis of *TET2* and *DNMT3A* exacerbates atherosclerosis and heart failure is proposed to be that mutated macrophages become pro-inflammatory, leading to the growth of arterial plaques and myocardial damage [36, 40–43]. Moreover, clonal hematopoiesis is frequently observed not only in the elderly but also in relatively younger patients who have undergone cancer treatment. In addition, therapy-related clonal hematopoiesis is a condition found in cancer survivors that may contribute to an increased risk of cardiovascular events [41]. In these cases, clonal hematopoiesis frequently detects DNA damage response genes such as protein phosphatase, Mg^2+/^Mn^2+^ dependent 1D, *PPM1D*; tumor protein p53, *TP53*; ataxia-telangiectasia mutated, *ATM*; and checkpoint serine-threonine kinase 2, *CHEK2* [44]. Recent experimental studies have reported that clonal hematopoiesis due to *PPM1D* and *TP53* genes exacerbates hypertensive heart failure and doxorubicin cardiomyopathy, respectively [39, 41, 45]. While previous reports on heart failure primarily focused on HFrEF, very recent studies have shown that clonal hematopoiesis is also associated with the pathology of HFpEF, garnering significant attention [40]. For instance, clonal hematopoiesis is thought to contribute to the formation of a distinct population of blood cells with acquired DNA mutations that are associated with an increased risk of heart failure, especially HFpEF [34, 37, 39, 40]. A study investigated the significance of clonal hematopoiesis in patients with HFpEF versus those without HFpEF [40]. The study found that *TET2*-mediated clonal hematopoiesis was enriched among patients with HFpEF, and that these patients had worse heart function and prognosis, especially those aged ≥ 70 years [40]. In the same study, the authors used a *Tet2* clonal hematopoiesis model mouse induced to develop HFpEF via high-fat diet and N_ω_-nitro-L-arginine methyl ester (L-NAME) stimulation. They reported significant diastolic dysfunction in the *Tet2* group, as demonstrated by echocardiography and catheter examinations [40]. Additionally, a large-scale epidemiological study also reported that clonal hematopoiesis is associated with HF, with a stronger association with HFpEF [39]. Additional studies in mice have revealed evidence for immune dysregulation in HFpEF and that genetic mutations associated with CHIP induce skewing of immune cells to a pro-inflammatory phenotype in vivo and in vitro [46, 47]. Inactivating *Tet2* and *Dnmt3* in mice has promoted angiotensin-II-induced cardiac dysfunction [46]. Furthermore, *Tet2* deficiency in hematopoietic cells in mice is associated with greater cardiac dysfunction resulting from elevated signaling of interleukin-1β [47]. Thus, clonal hematopoiesis, including medullary hematopoiesis, can be considered an important factor modifying heart failure, and the development of diagnostic and therapeutic approaches focusing on these aspects is highly anticipated.

## Extramedullary hematopoiesis in the spleen and heart failure

Extramedullary hematopoiesis is the term used to describe the process of hematopoiesis that occurs in organs other than the bone marrow [17, 48]. For example, during immune responses, the spleen or liver can serve as sites for extramedullary hematopoiesis [48]. It is important to point out that studies conducted with humans are lacking. Therefore, the data provided in this section are derived from research carried out on animals. Evidence from a mouse model indicates that extramedullary hematopoiesis in the spleen contributes to the risk of heart failure [48]. Another mouse model study showed that deletion of a G-protein-coupled receptor ALX results in inflammation, along with an increase of pro-inflammatory Ly6Chi C–C chemokine receptor type 2^+^ macrophages in the spleen and heart at a steady state, leading to an inflamed splenocardiac axis [49]. The authors suggested that ALX receptor deficiency may provide an experimental model that includes cellular and molecular mechanisms observed in HFpEF. Research on the impact of extramedullary hematopoiesis in the spleen on the risk of heart failure in humans is needed. Physiologic extramedullary hematopoiesis occurs in fetuses but not in adults [17]. However, when triggered by stress or disease, the spleen undergoes extramedullary hematopoiesis in adults [48]. Extramedullary hematopoiesis in the spleen can be a response to hematopoietic stress caused by microbial infections and diseases such as myelofibrosis [50], malaria [51], atherosclerosis [15], and osteopetrosis [52]. A recent study examined the effect of psychosocial stress on mice exposed to mild social defeat stress, with the authors observing severe extramedullary hematopoiesis in the spleen in mice exposed to social defeat stress [53].

## Spleen and heart failure

**Fig. 1:**
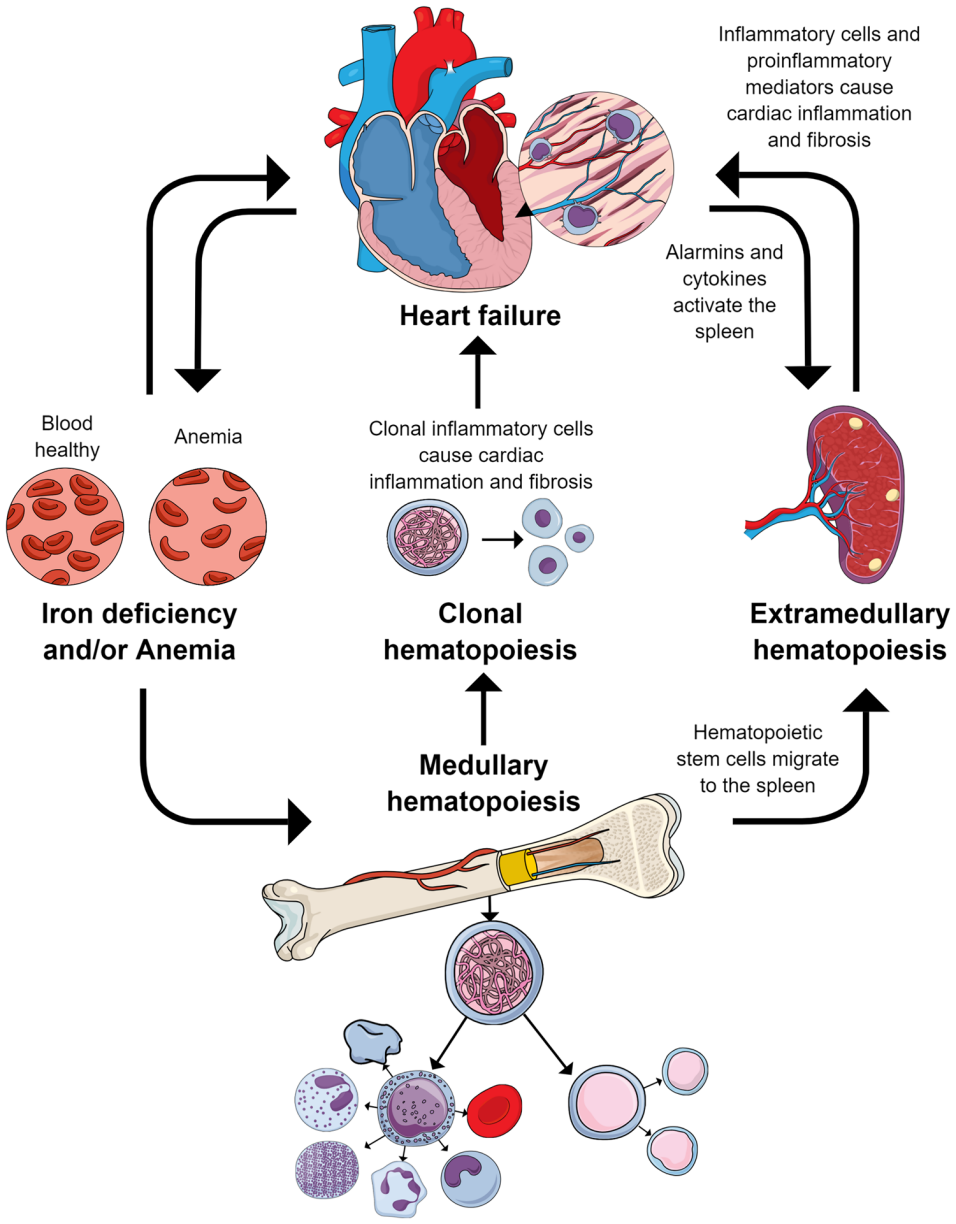
Model of the interplay of the heart, bone marrow, and spleen relating to heart failure, from the perspective of spleen-based extramedullary hematopoiesis

The links between the spleen and heart in heart failure, termed the cardiosplenic axis, were described in detail in a recent review by our group [54]. Of note, the causal directionality of the relationship between spleen size and heart failure may not always be apparent [54]. An interplay between chemical messengers sent out by the heart and received by the spleen may promote heart failure (Fig. 1). For example, in the infarcted heart, infiltrating neutrophils release alarmins that can activate splenic cells to produce immunoglobulin G; this accumulates in the atherosclerotic plaque, forming immune cell-immunoglobulin G complexes that promote inflammation [55, 56]. Additionally, an interaction exists among the sympathetic nervous system, bone marrow, and spleen in heart failure. The sympathetic nervous system is stimulated after myocardial infarction, which relays signals back to the bone marrow. This results in the release of hematopoietic stem cells, which travel to the spleen and undergo extramedullary hematopoiesis [57]. Monocytes produced via extramedullary hematopoiesis stimulated by the sympathetic nervous system go on to infiltrate atherosclerotic lesions, increasing the susceptibility of the lesions to rupture and leading to an increased risk of secondary myocardial infarction [57]. The spleen performs highly important hematological and immunological functions, and the structure and function of the spleen have been described elsewhere [54].

A recent review looked at the role of splanchnic circulation in the pathogenesis of heart failure, along with potential novel treatment options for redistributing blood volume in patients with heart failure, especially HFpEF [58]. The highly vascularized organs of the splanchnic compartment, which include the liver, spleen, and mesenteric vasculature, hold the majority of the body’s intravascular blood volume. Excess fluid redistribution from this compartment leads to increased cardiac filling pressures. This inappropriate regulation of blood volume distribution is suggested to be a factor contributing to the exercise intolerance seen in HFpEF [58]. The role of the sympathetic nervous system in modulating the capacitance and compliance of the splanchnic vascular bed via modulation of the greater splanchnic nerve, and greater splanchnic nerve ablation for volume management, have been proposed as a potential therapeutic intervention to increase unstressed blood volume. Studies have shown that right-sided greater splanchnic nerve ablation was safe and improved mostly subjective clinical metrics such as N-terminal pro-brain natriuretic peptide, health status, and 6-min walk distance (6MWD) in patients with HFpEF over 12 months [59, 60]. These findings suggest that splanchnic nerve modulation provides a strong foundation for research into this potential therapeutic target in HFpEF.

The outcome in patients who do not have a spleen or who have undergone a splenectomy is often poor. Although other organs such as the liver and bone marrow can take over most of the spleen’s functions, patients without a spleen have an increased risk of infections, given that the spleen plays a significant role in fighting infections [61]. A major contributor to poor outcomes after splenectomy is an increase in infections and infectious complications [62, 63]. Surgical removal of the spleen in humans can result in elevated low-density lipoprotein cholesterol, altered lipid values, and atherosclerosis [63, 64]. A 2016 study examining long-term outcomes after pediatric splenectomy [62] reported various findings, including a very low risk of portal vein thrombosis, the association of sepsis with underlying immunodeficiency, and a low recurrence rate of hematologic disorders. A study using a rat model found splenectomy to improve portal hypertension in both cirrhotic and non-cirrhotic models, while hypertension related to anemia and thrombocytopenia was improved in the non-cirrhotic model [65]. Isolated congenital asplenia, which is an extremely rare condition, has been described in a case report [66]. The patient was diagnosed with reactive thrombocytosis related to hyposplenism, with the authors suggesting vaccination against the main encapsulated bacteria in such cases, given the high mortality associated with such infections [66]. A 2014 study included 8149 USA veterans who underwent splenectomy with up to 27 years of follow-up. Those who underwent splenectomy had increased risks of infections, thromboembolism, coronary artery disease, and malignancies that persisted over 10 years after splenectomy [67]. Another study found that World War II servicemen who had undergone splenectomy because of trauma were 1.9 times more likely to die of ischemic heart disease [68]. While a splenectomy does not decrease the risk of heart failure, it may increase the risk of cardiac complications. Conversely, hypersplenism may be a cardiovascular risk factor for patients with heart failure [69]. Red blood cell destruction is a manifestation of hypersplenism and may contribute to the increased production of reactive oxygen species in heart failure progression. Table 1 shows clinical data that support the critical importance of the spleen for heart failure progression.

**Table 1:** Possible roles of the spleen on cardiac function in animal models

**Model**	**Macro-level mechanisms**	**Micro-level mechanisms**	**Outcome or findings**
**Murine model of heart failure **[117]	Splenomegaly and splenic tissue remodeling	More lymphocytic white pulp centers with large germinal centers and hypertrophy of the surrounding marginal zone Reduced mononuclear cell abundance in red pulp	Persistent mobilization of splenic pro-inflammatory monocytes to the heart
***Ccr2***^**−/−**^** mice underwent myocardial infarction **[18]		Increase in blood monocytes and decrease in splenic monocytes	The released monocytes did not accumulate in the ischemic myocardium because of the lack of the CCR2 chemokine
**Mice were splenectomized such that bone marrow and blood monocytes were preserved, then underwent myocardial infarction **[18]		Blood monocyte numbers increased in control but not in splenectomized mice	The spleen mobilizes monocytes after myocardial infarction
**CD45.1 splenectomized mice were given CD45.2 spleens by transplantation, and then underwent myocardial infarction **[18]		Donor blood monocyte numbers increased	Myocardial infarction triggered the release of splenic monocytes
***Atgr1a***^**−/−**^** mice underwent myocardial infarction **[18]		Splenic monocytes were not released efficiently. Few monocytes accumulated in the ischemic myocardium	Angiotensin II-angiotensin type 1 signaling contributes to the release of splenic monocytes
**Wistar rat heart donors, Sprague–Dawley rat recipients of heart transplantation with and without splenectomy **[118]	Sprague–Dawley rats with heart transplantation and splenectomy had delayed myocardial damage	Sprague–Dawley rats with heart transplantation and splenectomy had delayed inflammatory cell infiltration Splenectomy increased the rate of lymphocyte apoptosis	Sprague–Dawley rats with heart transplantation and splenectomy had significantly prolonged mean survival time of heart allografts
**Adult male C57BL/6J mice were splenectomized with intracerebral hemorrhage or underwent splenectomy alone **[119]	Splenectomy alone improved acute (7 days) or chronic (28 days) cardiac dysfunction, with increased left ventricular ejection fraction and left ventricular fractional shortening, and decreased cardiac fibrosis	Splenectomy alone exhibited decreased cardiac infiltration of immune cells and decreased expression of inflammatory factors and oxidative stress in the heart	Splenectomy alone did not induce acute (7 days) or chronic (28 days) cardiac dysfunction Mice with intracerebral hemorrhage and splenectomy had significantly improved acute and chronic cardiac function compared with mice that received intracerebral hemorrhage alone

*Atgr1a* angiotensin receptor 1a, *Ccr2* C–C chemokine receptor type 2

## Iron metabolism in heart failure

Heart failure is frequently associated with iron deficiency with or without anemia [70]. Iron is essential for the optimal functioning and survival of living organisms. Iron deficiency leads to mitochondrial dysfunction, aberrant enzyme activity, abnormal transport of structural proteins, and apoptosis [71]. Moreover, the ability of organs and tissues to function properly is impaired, along with decreased exercise capacity, work functioning, and cognitive performance, and increased morbidity and mortality [71].

Two types of iron deficiency have been proposed: absolute iron deficiency and functional iron deficiency. Absolute iron deficiency is defined by severely reduced or absent iron stores in bone marrow, liver, and spleen [71]. Functional iron deficiency is defined by normal or increased total body iron stores that are unavailable for incorporation into erythroid precursors for erythropoiesis owing to increased levels of hepcidin [71]. Patients with heart failure and iron deficiency have lower levels of iron in the spleen and liver [72]. The spleen plays an important role in the circulatory system, i.e., removal of aged or damaged red blood cells. Macrophages that reside in the red pulp of the spleen, characterized by the expression of CD163, mediate the turnover of aging red blood cells [25]. Furthermore, the spleen participates in red blood cell turnover by inducing hemolysis [25]. Aging red blood cells undergoing hemolysis in the spleen release hemoglobin to the environment; this may bind to the hemoglobin haptoglobin scavenger receptor, CD163, which is highly expressed in the spleen [73]. These findings provide evidence for the important role of the spleen in efficient iron recycling from senescent red blood cells.

Myocardial iron deficiency in heart failure is accompanied by decreased mitochondrial functions, diminished activity of citric acid cycle enzymes, and reduced expression of reactive oxygen species protective enzymes, suggesting that myocardial iron deficiency may contribute to myocardial dysfunction in heart failure [74]. Patients with heart failure and iron deficiency showed lower levels of iron in the spleen and liver, with a trend toward lower iron content in the heart’s septum [71]. Furthermore, iron levels in the left ventricle were significantly decreased in failing hearts compared with those in non-failing hearts [74]. These effects of iron deficiency can be reversed with therapy. After intravenous iron therapy, iron levels increased in the left ventricle and spleen [72, 75].

## Anemia and heart failure

Iron deficiency is the most common cause of anemia in patients with heart failure [76]. Mortality is higher in patients with heart failure and anemia than in patients with heart failure without anemia, even after adjustment for potential confounders [77]. A study in a Swedish heart failure registry explored the relationships between anemia and heart failure across the ejection fraction spectrum [78]. Anemia prevalence was the highest in HFpEF; anemia was independently associated with a similar risk of death regardless of ejection fraction but with a higher risk of heart failure hospitalization in HFpEF and HFmrEF than in HFrEF [78].

Renal dysfunction and chronic kidney disease are common complications of heart failure. The interaction among anemia, congestive heart failure, and chronic kidney insufficiency has been described as a vicious cycle termed cardio-renal anemia syndrome [79]. In chronic kidney disease and other conditions with impaired renal function, the production of erythropoietin, which stimulates the production of blood cells, is reduced, causing anemia [80]. Production of erythropoietin is regulated by hypoxia-inducible factor (HIF) [81]. Inhibitors of HIF prolyl-hydroxylase (HIF-PH) have recently been used to treat renal anemia and have improved anemia caused by chronic kidney disease and other conditions [80, 82]. Findings from a recent study by Nakamura et al. suggest that HIF-PH inhibitors may be safe and effective for treating renal anemia in patients with heart failure [83]. The FAIR-HF trial showed that treatment with intravenous ferric carboxymaltose in patients with chronic heart failure and iron deficiency with or without anemia improved symptoms and quality of life [84]. Furthermore, intravenous iron therapy in patients with heart failure with or without anemia demonstrated improvement in subjective symptoms, quality of life, and the 6MWD test [84]. A meta-analysis of data from several clinical trials indicated that treatment with intravenous ferric carboxymaltose is associated with a reduction in recurrent hospitalizations in patients with heart failure and iron deficiency [85]. Treatment of patients with acute non-compensated heart failure and iron deficiency with ferric carboxymaltose was safe and reduced the risk of hospitalization for heart failure [86]. In addition, patients with chronic heart failure treated with intravenous iron alone reported improvements in their subjective symptoms and an improved 6MWD with increased hemoglobin [87]. Sodium-glucose cotransporter 2 (SGLT2) inhibition with empagliflozin may stimulate erythropoiesis and improve functional iron deficiency in patients with type 2 diabetes and heart failure [88]. The DAPA-HF trial examined the effect of SGLT2 inhibitors on outcomes in patients exhibiting a wide range of HFrEF. The study demonstrated that the impact of dapagliflozin on anemia reversal remained steady, irrespective of whether iron deficiency was present or absent initially. The study indicated a potential therapeutic synergy between iron supplementation and SGLT2 inhibition, which could be beneficial for some patients with HFrEF to prevent iron deficiency and to treat anemia [89]. In addition, the 2022 American College of Cardiology/American Heart Association/Heart Failure Association of America Guideline for the Management of Heart Failure [21] recommends SGLT2 inhibition for patients because it has been shown to decrease the risk of hospitalization for heart failure and cardiovascular mortality in patients with heart failure, regardless of whether they have type 2 diabetes. This benefit applies for both patients with HFrEF and those with HFpEF [90]. However, a recent review pointed out that SGLT2 inhibitors have been shown to decrease hepcidin and ferritin and increase transferrin receptor protein, which is suggestive of worsening iron deficiency [91]. That review advocates for additional studies of the efficacy and safety of concurrent use of SGLT2 inhibitors and intravenous iron in patients with heart failure.

## Links between inflammation and heart failure

The inflammasome (a part of the innate immune system) has been investigated in heart failure and has been shown to play a role in pathological changes in the heart, such as hypertrophy, fibrosis, and cell death [92, 93]. Multiple studies have highlighted the importance of sterile inflammation in heart failure. Sterile inflammation occurs in the absence of infection and involves the secretion of inflammatory cytokines and the recruitment of innate immune cells, such as neutrophils and monocytes/macrophages [94, 95]. Inflammation leads to an increased release of hepcidin from the liver, which downregulates ferroportin, resulting in (1) reduced transport of dietary iron intake from the inside of mucosal cells to the small intestine to the bloodstream [96] and (2) reduced release of recycled iron from the macrophages in the spleen and liver to the bloodstream, which in turn may lead to iron deficiency [25].

Immune dysregulation plays a role in heart failure via extramedullary hematopoiesis occurring in the spleen. A study in mice has shown that the spleen is a major site of macrophage production after myocardial infarction [97]. The authors screened spleen mRNA levels after myocardial infarction and found a marked increase in the expression of interleukin-1β [97]. Of note, pro-inflammatory cytokines such as tumor necrosis factor-α and interleukin-6 have been found to have a direct effect on the bone marrow and may be involved in the mechanism of anemia in chronic disease [98]. Ischemic injury to the heart leads to the replacement of the cardiac resident macrophages in the infarct zone with monocyte-derived macrophages from the bone marrow and spleen, which may result in a pro-inflammatory environment [99]. However, a study provided evidence that splenic leukocytes and macrophages promote left ventricle healing by generating specialized proresolving mediators, such as lipoxins, resolvins, protectin, and maresin, which are enzymatically produced during the resolution of inflammation [100, 101]. Heart failure induces monocytopoiesis in the bone marrow and in the spleen [57, 102]. The PREFER-HF study will comprehensively evaluate the relationship between clinical characteristics, genomic, proteomic, and metabolomic data, along with imaging information and clinical outcomes in a US cohort receiving ambulatory care [103]. Data are expected to be reported in approximately 5 years.

## Immune cell mobilization in heart failure

The spleen may compensate for decreased bone marrow function associated with heart failure by increasing its own function [57]. A study on the effects of social stress in mice revealed the possibility that splenic erythropoiesis may partially alleviate the anemia associated with stressful situations (inflammation) [104]. The phenotype of cardiac resident macrophages differs from blood-derived macrophages from the spleen [98]. Cardiac macrophages have an M2-like gene expression signature, suggesting that these macrophages play an immunomodulatory function in the heart [105]. Cardiac resident macrophages are protective for the heart, while spleen-derived macrophages help heal the injured myocardium and promote pathology [106]. Elevated leukocyte counts have been shown in patients with heart failure after myocardial infarction [100]. In addition, the spleen has been shown to provide a steady flow of leukocytes to the left ventricle after myocardial infarction [100]. A study looked at leukocyte trafficking and specialized proresolving mediators in the spleen and in the left ventricle after myocardial infarction [100]. The study’s findings suggested that leukocytes mobilize from the spleen to the heart and generate lipids that aid in resolving the inflammation after myocardial infarction [100]. Recent findings have described an iron-dependent form of cell death termed ferroptosis in cardiac injury resulting from both chemotherapy and ischemia/reperfusion [107]. Ferroptosis is distinct from both apoptosis and necroptosis and can be halted or reversed with iron chelation [107, 108]. Neutrophils are thought to impair myocardial repair and contribute to heart failure progression via the generation of damaging cytokines and reactive oxygen species and the release of enzymes stored in secretory granules [109]. Neutrophils may have cardioprotective properties such that the generation of reactive oxygen species by neutrophils may stimulate the differentiation of reparative macrophages [110].

## Clinical implications and future directions

Further exploration of the interplay between the heart, spleen, and bone marrow, from the viewpoint of extramedullary hematopoiesis, is warranted. Many of the findings have been developed using mouse models and may not be representative of patients with cardiac diseases. In fact, the absence of a strong HFpEF in vivo model restricts our understanding of the pathophysiology of HFpEF. No existing in vivo model encapsulates all the hemodynamic features of HFpEF [111, 112]. This presents a significant challenge in our understanding and research of this condition. Animal models of HFpEF are under active investigation. A recent study created several mouse models of HFpEF by combining three independent pathological stressors (increased calcium ion influx, high-fat diet, and L-NAME). The study showed that histone deacetylase 6 *(Hdac6)* deletion in mice slows HFpEF progression, and that a specific HDAC6 inhibitor, TYA-018, reverses existing cardiac hypertrophy and diastolic dysfunction in mouse models of HFpEF [113], suggesting that HDAC6 inhibition may be a therapeutic approach for HFpEF.

In humans, two clinical trials have explored the effect of the SGLT2 inhibitor empagliflozin in HFpEF [114–116]. The placebo-controlled EMPEROR-Preserved trial evaluated the effect of empagliflozin on a primary outcome of a composite of cardiovascular death or hospitalization for heart failure, reporting that empagliflozin reduced the risk of the primary outcome in patients with HFpEF, regardless of the presence or absence of diabetes [116]. The EMPERIAL-Preserved trial evaluated the effects of empagliflozin on exercise ability and patient-reported outcomes in patients with HFpEF, with or without diabetes [115]. While the effect of empagliflozin on the primary outcome—6MWD—was neutral, the authors noted that empagliflozin was well tolerated in HFpEF patients, both with and without diabetes.

Overall, further studies are needed to better understand the effects of extramedullary hematopoiesis on heart disease, and further research in animal models of HFpEF and of therapeutic approaches in patients with HFpEF is welcome. Table 2 provides proposed hypotheses from the viewpoint of extramedullary hematopoiesis in the spleen in heart failure.

**Table 2:** Proposed hypotheses of the interplay of the heart, spleen, and bone marrow, from the perspective of spleen-based extramedullary hematopoiesis, relating to heart failure (HFpEF)

**Mechanisms of heart failure including HFpEF in the context of the interplay of the heart, spleen, and bone marrow**	
Hypothesis	Evidence to support the hypothesis
**Genetic level**	
**In clonal hematopoiesis, as a consequence of genetic damage, there is an increase in abnormal red blood cells**	A higher distribution of red blood cells is associated with increased mortality [120]
**Molecular and cellular level**	
**Changes in the number or balance of splenic macrophages occur**	Research on mice has shown that the splenic monocytes increase their motility, exit the spleen, and accumulate in the injured myocardial tissue [18] In myocardial infarction, stimulation from the sympathetic nervous system results in the release of hematopoietic stem and progenitor cells from bone marrow niches. The progenitors then localize to the spleen to induce a prolonged increase in monocyte production [57]
**Changes in the function of splenic macrophages occur**	Based on the finding that injury to the heart leads to the replacement of resident cardiac macrophages with monocyte-derived macrophages from bone marrow and spleen [99], we can speculate that the function of splenic macrophages may change in heart failure Research in mice with ischemic heart failure has shown an increased proportion of CCR2^+^ splenic macrophages and a decreased proportion of resident CCR2^−^ macrophages in heart failure versus sham-operated hearts at 16 weeks after myocardial infarction or sham operation [117]. Mice with ischemic heart failure consistently demonstrate splenomegaly and splenic tissue remodeling compared with sham-operated mice [121]
**Iron metabolism is imbalanced, and anemia develops**	Iron levels in the spleen and liver are reduced in patients who have heart failure and iron deficiency, which suggests that treating iron deficiency might be beneficial [72, 75] In advanced heart failure, the iron content in the myocardium is reduced and the functions of the mitochondria are compromised [74]
**Iron deficiency leads to mitochondrial dysfunction and possible myocardial impairment**	Decreased iron intake leads to inflammation, increased levels of hepcidin, impaired iron release from tissues, and decreased absorption of iron [22, 74]
**Inflammation may play a role in causing iron deficiency**	During conditions in which the hepcidin level is abnormally high, such as inflammation, serum iron falls because of iron trapping within macrophages and liver cells and decreased gut iron absorption [63]. The link between inflammation/infection and liver production of hepcidin is attributed to interleukin-6, produced at sites of infection/inflammation [71]
**Anemia worsens the prognosis of heart failure, creating a vicious cycle**	Chronic kidney disease may cause anemia owing to decreased production of erythropoietin, which is responsible for the stimulation of blood cell production [80] Anemia may be caused by a mechanism involving pro-inflammatory cytokines such as tumor necrosis factor-alpha and interleukin 6, which directly affect the bone marrow [98]
**Chronic inflammation and progressive fibrosis can lead to decreased cardiac function**	Macrophages in the heart have an M2-like gene expression pattern, indicating that they may have a role in regulating the immune response within the heart [105] Research in mice with diastolic dysfunction shows a higher myocardial macrophage density resulting from the recruitment of monocytes and increased hematopoiesis in the bone marrow and spleen [120]
**The balance of immune cells produced by the spleen and bone marrow may promote heart failure**	Research on mice has indicated that, following myocardial infarction, the spleen becomes a primary location for the production of macrophages [97, 122] When the heart experiences ischemic injury, the macrophages in the infarct zone are replaced by monocyte-derived macrophages from the bone marrow and spleen. This can create a pro-inflammatory environment [99]
**Tissue and organ level**	
**A loss of bone marrow niches occurs in aging and disease**	When triggered by stress or disease, the spleen might serve as an alternate tissue source for hematopoiesis [123]
**An interaction of the sympathetic nervous system, bone marrow, and spleen occurs in heart failure**	Stimulation of the sympathetic nervous system after myocardial infarction sends signals to the bone marrow, resulting in the release of hematopoietic stem cells, which travel to the spleen and undergo extramedullary hematopoiesis [50]
**The spleen undergoes compensatory changes in function that are cardioprotective**	The spleen releases interleukin-10, which is involved in cardioprotection [124] Research in mice has shown that splenic erythropoiesis may partially alleviate the anemia associated with heart failure, which is a stress situation, especially for inflammation, and may influence its pathogenesis and prognosis [104]
**The spleen undergoes compensatory changes in function that promote heart failure**	Spleen-derived macrophages help to heal the injured myocardium, but they promote pathology [106]
**Decreased activity or absence of the spleen promotes heart failure**	Research on the impact of splenectomy on humans suggests that the spleen can regulate the movement of macrophages into coronary arteries and the development of atherosclerosis [63]
**Iron retrieval in the spleen is impaired**	Reduced levels of iron are observed in the spleen and liver of patients with heart failure [72, 75] After intravenous iron therapy, iron levels increased in the left ventricle and spleen [72, 75]

The table provides the proposed hypotheses arising from the concept of the interplay of the heart, spleen, and bone marrow from the viewpoint of spleen-based extramedullary hematopoiesis in heart failure, including HFpEF. The information is organized into levels, including genetic, molecular, cellular, and tissue and organ levels. The table also encompasses aspects such as iron metabolism, anemia, inflammatory cytokines, and immune cells. Potential therapeutic targets or approaches are also suggested at various levels where relevant

*HFpEF* heart failure with preserved ejection fraction, *CCR2* C–C chemokine receptor type 2

## Conclusions

Medullary hematopoiesis plays a role in heart failure. Bone marrow-derived circulating endothelial progenitor cells are associated with cardiovascular risk factors, including increased age, diabetes, smoking, and hypertension. Clonal hematopoiesis, which occurs in the bone marrow, especially in older adults, is consequently involved in the development of cardiovascular diseases, including heart failure. This is owing to the abnormal immune cells produced. The spleen serves as an important source of immune system cells, and immune dysregulation plays a role in heart failure through extramedullary hematopoiesis occurring in the spleen. In heart failure, emergency hematopoiesis occurs in the spleen, i.e., immune cells are also produced (monocytes, macrophages, neutrophils), which are mobilized to the heart and the rest of the body. Therefore, we speculate that extramedullary hematopoiesis may be a key factor in the development of heart failure, especially HFpEF. Extramedullary hematopoiesis in the spleen is a compensatory change in the pathogenesis of heart failure but may also be a mechanism for the progression and exacerbation of chronic heart failure (especially HFpEF). This results from the repeated increase in metabolic activity in the spleen, immune cell production, mobilization to the heart, and concomitant cytokine production. Ultimately, this leads to systemic chronic inflammation, as well as to an imbalance of immune cells (macrophages) in the heart, resulting in chronic inflammation and progressive fibrosis, potentially causing decreased cardiac function.

## Data Availability

Not applicable.
